# Identification of polymorphic alleles in TERC and TERT gene reprogramming the telomeres of newborn and legacy with parental health

**DOI:** 10.1016/j.sjbs.2023.103897

**Published:** 2023-12-12

**Authors:** Sadia farrukh, Saeeda Baig, Rubina Hussain, Rehan Imad, Ome kulsoom, Mehreen Yousaf Rana

**Affiliations:** a Department of Biochemistry, Ziauddin University, Karachi, Pakistan; b Department Gynecology and obstetrician, Ziauddin university and hospitals, Karachi, Pakistan; c Department of Molecular medicine, Ziauddin University Karachi, Pakistan; d Department Gynecology and obstetrician, Ziauddin hospital, Karachi, Pakistan; e Department of Community Health Sciences, The Agha Khan University, Karachi, Pakistan

**Keywords:** Telomere, Telomerase, TERC, TERT, Polymorphism, Gene, Diabetes, Hypertension, Gestational Diabetes Mellitus (GDM), Preeclampsia

## Abstract

Telomere and telomerase genes (TERC and TERT) highlighted many novel genetic polymorphisms related to common diseases. This study explored the polymorphic alleles of TERC and TERT gene in parents-newborn (triad) and its association with telomere length (TL) and parental diseases (mother: Gestational Diabetes Mellitus (GDM), Preeclampsia, fathers: Diabetes, Hypertension). In this cross-sectional study, the blood samples (n = 612) were collected from parents-newborn triad (204 each) for TL (T/S ratio) quantification by using qPCR, and gene (TERC and TERT) polymorphism was detected by Sanger sequencing. The correlation analysis was used to find an association between paternal TL (T/S ratio) and newborn TL. The multivariate linear regression was applied to determine the effect of parents genes and diseases on newborn TL. A positive association (r = 0.42,0.39) (p < 0.0001) among parents and newborn TL was observed. In the diseased group, both TERC (rs10936599) and TERT (rs2736100) genes had a high frequency of allele C in newborns (OR = 0.94, P = 0.90, OR = 4.24, P = 0.012). However, among parents, TERT gene [Mother CC (B = 0.575; P = 0.196), Father CC (B = -0.739; P = 0.071)] was found significant contributing factor for Newborn TL. Diseased parents with T/T and A/C genotypes had longer newborn TL (2.82 ± 2.43, p < 0.022; 1.80 ± 1.20, p < 0.00) than the C/C genotype. Therefore, the study, confirmed that major allele C of TERC and TERT genes is associated with smaller TL in diseased parents-newborns of the targeted population.

## 1 Introduction

Telomere, a biological marker of a cell is highly conserved having hexameric repeats (TTAGGG), and is maintained by a ribonucleoprotein enzyme telomerase. The telomere length (TL) is maximum at birth gradually shortens in every cell division, and undergoes senescent or initiates apoptosis. Among, all cells telomere length of leukocytes has been considered a standard indicator for the risk of diseases. Telomere is maintained by telomerase, which is expressed in stem cells, germ cells, cancer cells, and proliferating cells like human T and B cells (Razgonova et al., 2020). In addition, the role of telomerase is also seen in several non-dividing cells “neurons and cardiac myocytes” to protect the cells from oxidative damage (Richardson et al., 2012, Iannilli et al., 2013, Li et al., 2021). Moreover, telomeres elongation by telomerase and different proteins takes place during the “S” phase of the cell cycle (Jafri et al., 2016).

For telomere maintenance two major genes, “TERC (telomerase reverse component) and TERT (Telomerase reverse transcriptase)” are required by the cell. The TERC gene is expressed mostly in normal human cells, highlighting that it may have other roles in the body such as accelerating secretion of the inflammatory cytokines (Liu et al., 2019). The other gene, TERT, is a telomerase catalytic subunit to which TERC adds GGTTAG repeats to chromosome ends and acts as a TERT template. Both genes together make the telomerase complex (Dratwa et al., 2020).

The TERC and TERT gene single nucleotide polymorphism (SNPs) may depict the telomeres length variation or genetic inheritance pattern. In this respect, it has been found that the reprogramming of newborn TL in utero may influence telomere molecular longevity (Farrukh et al., 2019). Many Genome-wide association studies (GWAS) on TERC and TERT act as powerful tools to illuminate the genetic role of complex disorders and highlight the novel findings of the genetic polymorphism related to many common diseases and traits (Liu et al., 2014, Mohamadkhani et al., 2019, Wolpin et al., 2014, Zhang et al., 2017, Scarabino et al., 2017, Scarabino et al., 2019, Li et al., 2021). The different SNPs of TERC like rs3772190, rs12696304, rs16847897, and rs10936599 are all located downstream or upstream of TERC and associated with TL variations (Codd et al., 2010, Maubaret et al., 2013). The TERC variant rs10936599 is associated with TL attrition and is located upstream of TERC in the “Myoneurin (MYNN)” gene that controls the expression of distinct genes (Sethi et al., 2020). The second gene TERT has many SNPs associated with both LTL and lifespan like MNS16A (minisatellite, regulating human telomerase expression) on exon 16, rs2853691 on intronic region, rs33954691 on exon 14 and rs2736100 on intron 2. (Liu et al., 2014, Soerensen et al., 2011).

Premature aging, cardiovascular disease, and diabetes result in genetic variation in newborns that may lead to dysfunctional or inactive proteins involved in TL regulation or DNA damage response which also involve repair pathways. Studies have highlighted that telomere biology and telomerase genes (TERC and TERT) have a role in age-related disease pathogenesis like diabetes and hypertension (Turner et al., 2019, Sethi et al., 2020, Cheng et al., 2020). Therefore, Cellular aging, inflammation, and oxidative stress lead to telomere length alteration due to telomerase activity (Liu et al., 2019).

There is a dearth of data regarding the effect of TERC and TERT polymorphism on TL variation in parents-newborns and the impact of parents' diseases on newborn telomere genetics. Moreover, how this impact on telomeres is transmitted to the next generation is not known. Thus, the respective study can be useful for the detection of altered telomere genetics leading to comorbidity, mortality, or aging in parents and their newborns. Newborn telomere length can be a detection marker of inherited remodeled genetics in newborns and its alteration and polymorphism can be used for prediction of risk of different diseases in newborns and later life. Therefore, this study aimed to associate the polymorphisms of telomerase gene (TERC and TERT) in parents-newborn (triad) with telomere length and parental diseases (mother: Gestational Diabetes Mellitus(GDM) and preeclampsia, fathers: Diabetes and Hypertension).

## 2 Materials and Methods

In this study, n = 612, parents-newborn (triad) (204/each) were enrolled from “Ziauddin Hospitals, Karachi, Pakistan” from September 2021-June 2022. This was a cross-sectional study approved by the “Ziauddin University”, Ethics Review Committee (Ref No. 3950721SFBC). The females aged 18 to 35 years and with a gestational age > 35 weeks (ultrasound data) and their husbands aged 18 to 45 years were included in the study after taking informed consent. Mothers with diseases like Gestational Diabetes Mellitus(GDM) and preeclampsia and fathers with diabetes and hypertension were included for further study. Whereas, parents with any cancer, tobacco (smoker, chewer) users, and drugs were excluded from the study. The performa was used to record laboratory investigations and parents' history of diseases. The venous blood of the parents (5 ml) and umbilical venous cord blood (5 ml) was collected in EDTA (ethylenediaminetetraacetic acid) tubes and then stored at 4 °C. The DNA Blood Mini Kit (Qiagen, catalog number 51306, Germany) was used to extract DNA and stored at −80 °C. The DNA concentration and quality were measured by a Multiskan Sky spectrophotometer (Thermoscientific, USA).

The leukocyte telomere length (LTL), was quantification by qPCR following Cawthon., multiplex method (Cawthon., 2009) The blood pool (four healthy females and males) was used as reference DNA/ standard in all runs of the PCR reaction by using 5 dilutions from 150 to 1.85 ng for formation of a standard curve. Then the experimental DNA (parents-newborn (cord) was quantified. The qPCR reaction (25 µl) included: 11 µl of PCR master mix (Maxima Syber green, Catalog No. K0221 Thermoscientific, USA), 1 µl of each Primer(1 μM) of both telomere and Beta globin gene (single copy gene) and 10 µl of DNA (5–150 ng). The Agilent thermal cycler (Agilent, USA) was programmed for PCR reaction according to the recommendations (Cawthon, 2009), and data collection was done by software (AriaMx System Software version 17.1) and generated two “standard curves” one for the telomere signal at 74 °C and one for the beta-globin signal at 88 °C. In each qPCR experiment, a negative control was run in each reaction. All samples were analyzed in triplicate. Good linearity (R^2^ > 0.99) and efficiency(>90 %) were maintained in all reactions. For qPCR run quality and primer specificity, amplification curves and melt curves were observed. The T/S ratio was calculated for the quantification of telomere length. The T/S ratio of each sample was calculated as previously done (Cawthon., 2009). The average of three values of the T/S ratio was used for the TL calculation.

After qPCR, the participants were divided into two groups: healthy (n = 16) and diseased (n = 32)**.** Then Sanger sequencing of the selected samples (n = 48) was performed for SNPs of TERC and TERT genes. The SNPs were selected due to their association with telomeres and had a > 5 % minor allele frequency (MAF) in the targeted “Pakistani Population” according to the “1000 genome database”. The gene loci amplified (TERC: 617 base pairs, TERT: 544 base pairs) by conventional PCR using the following set of Primers. The primer for TERC and TERT gene amplification was: TERC; Forward primer 5’AAGCGTCAGGTTTTGCTGTG3’: Reverse primer 5‘TTGCTGTGAAGACTACTGACTAG3’, TERT; Forward:5′CTCGGAGCCTCATCCTTTGT3’;Reverse:5’TCTCAGGCATCTTGACACCC3’ (Synbio Tech, USA). The PCR reaction mixture of 50 μl included: 25 μl PCR master mix (DreamTaq, catalog Number: K0181, Thermo Scientific, USA), 2 μl of each primer (1 μM), 10 μl of DNA, and 11 μl of water. The thermal cycling conditions were set as mentioned in a previous study (Farrukh et al., 2022). Negative control was also run in all the PCR reactions. The ExoSap-IT PCR Product Cleanup kit(catalog no. 78200, Thermofischer, USA) was used for PCR product purification and the Big Dye Terminator Sequencing Kit (Catalog No. 4337456, Thermofischer, USA) was used for sequence analysis by SeqStudio Genetic Analyzer (Thermofisher Scientific, USA). Sequencing instrumentation software was used to detect, filter, and remove unacceptable nucleic acid sequence data to identify base-specific quality scores of targeted SNPs. The data that passed the Quality Control were then used for sequence analysis by another bioinformatics analysis software (Mega X).

### 2.1 Statistical analysis

The SPSS (version 24) was used for data analysis. The spearmen correlation was used to find the association between paternal TL and newborn TL. The Kruskal-Wallis test was applied for mean differences among allele groups and telomere length. The multivariate regression analysis was used to study the association between newborn TL (T/S ratio) and diseases and parents' TL and TERC and TERT genotypes. The Software “GraphPad Prism” was used to prepare graphs. The 1000 genome project (https://www.ncbi.nlm.nih.gov/projects/SNP) was used for the identification of different TERC and TERT gene SNP in the targeted region. The software “Mega X” was used for the sequencing analysis and SNP detection. The online software “*SHEsis:* http://analysis.bio-x.cn/myAnalysis.php” analyzes the genotype and allele frequency distribution. The p < 0.05 was considered statistically significant in all the tests.

## 3 Results

**Fig. 1:**
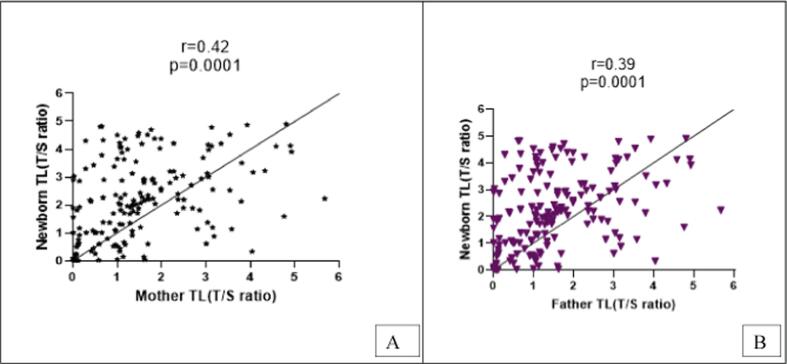
(A-B): Correlation between parents and newborn Telomere length(TL). Positive correlation between mother- newborn TL (r = 0.42, p = 0.0001) (4A) and father-newborn TL (r = 0.39, p = 0.0001) (4B).

**Fig. 2:**
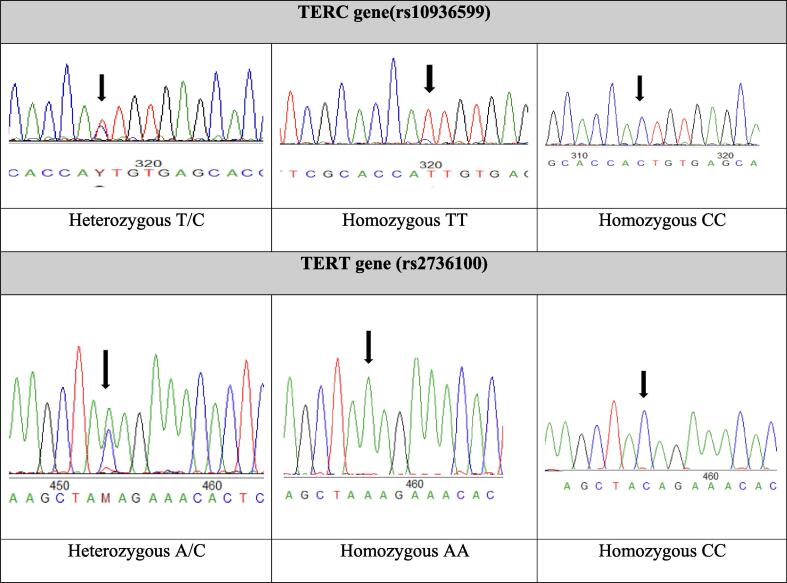
Single nucleotide polymorphism(SNP) of TERC and TERT genes. SNPs highlighting homozygous and heterozygous alleles (black arrows) of TERC and TERT genes. Sequences published on NCBI with accession numbers. **rs10936599**:OP046318-OP046360 https://www.ncbi.nlm.nih.gov/nuccore/OP046318**rs2736100**: OP081479-OP081528 https://www.ncbi.nlm.nih.gov/nuccore/OP081479. Red bars: Diseased, Green bars: Healthy, ns: no significant. (For interpretation of the references to colour in this figure legend, the reader is referred to the web version of this article.)

A total of 612 participants, parents-newborn triad (204/each) were recruited in the study. The positive association (r = 0.42,0.39) between parents and newborn Telomere length (TL) showed significant results (p=<0.0001), however, the mother had more effect than the father (Fig. 1A, 1B). The allele and genotypes frequency distribution of the targeted SNPs of telomerase genes TERC (rs10936599) and TERT (rs2736100) genes showed the C allele had the highest frequency in the triad (TERC: 67 %, 62.2 %,62.2 %, TERT: 70.4 %, 61.2 %,70.4 %). In Fig. 2 heterozygous and homozygous alleles of both TERC and TERT genes are highlighted.

**Fig. 3:**
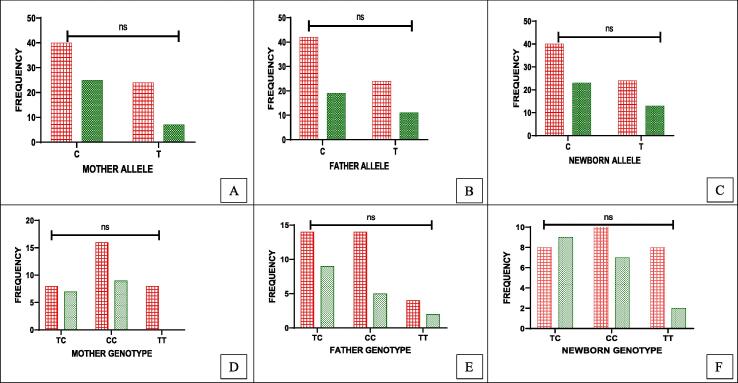
(A-F): Distribution of TERC (rs10936599) allele and genotypes among parents-newborns (triad) in healthy and diseased groups. 3(A-C): Allele C with high frequency in diseased parents and their newborns. 3D: Diseased mothers with high CC/TT 3E: CC/TC high in diseased fathers 3F: CC/TT high in newborns to parents with diseases. Red bars: diseased, Green bars: healthy.* significant results. (For interpretation of the references to colour in this figure legend, the reader is referred to the web version of this article.)

Distribution of the triad into healthy and disease groups showed that TERC gene polymorphic allele (C/T) distribution among the triad did not show significant results: Mother (OR = 0.46, P = 0.17); Father OR = 0.60, P = 0.60); Newborn (OR = 0.94, P = 0.90) (Table 1). Allele C was more frequent in both the diseased and healthy groups among the triad samples whereas, minor allele T was seen less frequently (Fig. 3A-C) (Table 1). In newborns, according to Hardy Weinberg Equilibrium (HWE) insignificant results were observed (p = 0.60, p = 0.72) (Table 2).

**Table 1:** Distribution of the allele frequencies of TERC and TERT genes in diseased and healthy triad.

**Variables (TERC)**	**Mother(n = 48) n(%)**		**Father (n = 48) n(%)**		**Newborn (n = 48) n(%)**	
**Allele**	**C**	**T**	**C**	**T**	**C**	**T**
**Diseased**	40(62.5)	24(37.5)	42(65.6)	22(34.4)	40(62.5)	24(37.5)
**Healthy**	25(78.1)	7(21.9)	19(59.4)	13(40.6)	23(63.9)	13(36)
**Hazard/Odds ratio**	0.466		0.60		0.94	
**95 % CI**	0.155 ∼ 1.40		0.473 ∼ 3.60		0.35 ∼ 2.52	
**X^2^**	1.87		0.26		0.01	
**P value**	0.17		0.60		0.90	
**TERT Allele**						
**Allele**	**C**	**A**	**C**	**A**	**C**	**A**
**Diseased**	42(65.6)	22(34.4)	34(53.1)	30(46.9)	38(59.4)	26(40.6)
**Healthy**	27(84.4)	5(15.6)	26(81.2)	6(18.8)	31(86.1)	5(13.9)
**Hazard/ Odds ratio**	2.82		3.82		4.24	
**95 % CI**	0.85 ∼ 9.40		1.23 ∼ 11.80		1.30 ∼ 13.79	
**X^2^**	3.0		5.74		6.22	
**P value**	0.08		0.01		0.012	

*SHEsis*, software analyses for genetic association at polymorphism loci.

**Table 2:** Distribution of the genotype distribution of TERC and TERT gene in diseased and healthy triads.

**Variables**		**Mother n(%)**		**Father n(%)**		**Newborn n(%)**	
**TERC Genotype n =** 48		**Diseased n = 32**	**Healthy n = 16**	**Diseased n = 32**	**Healthy n = 16**	**Diseased n = 32**	**Healthy n = 16**
**T/C**		8(25)	7 (43.8)	14 (48.3)	9 (56.2)	8 (25)	9 (0.50)
**C/C**		16 (50)	9 (56)	14 (48.3)	5 (31.2)	16 (50)	7 (38.9)
**T/T**		8(25)	0(0)	4 (12.5)	2 (12.5)	8 (25)	2(11)
**X^2^**		4.87		0.58		2.54	
**P VALUE**		0.87		0.74		0.27	
**HWE**	**X^2^**	3.48	1.25	0.14	0.44	3.48	0.12
	**P value**	0.06	0.26	0.90	0.50	0.60	0.72
**TERT Genotypes n =** 48							
**A/C**		22 (68.8)	5 (31.2)	10 (31.2)	6 (37)	18 (56.2)	5 (27)
**C/C**		10 (31.2)	11(68.8)	12 (37.5)	10 (65)	10 (31.2)	13 (72)
**A/A**		0(0)	0(0)	10(31.2)	0(0)	4(12.5)	0(0)
**X^2^**		4.5		6.09		6.60	
**P VALUE**		0.03		0.04		0.03	
**HWE**	**X^2^**	4.39	0.54	2.22	0.85	0.44	0.46
	**P value**	0.79	0.45	0.13	0.35	0.50	0.49

X^2^:Chi^2,^ HWE: Hardy-Weinberg equilibrium test, *SHEsis*, software analyses for genetic association at polymorphism loci.

In Fig. 3(D-F) and Table 2, the comparison of different frequencies of genotypes between diseased and healthy highlighted that mothers had T/T (25 %) genotype, only in a diseased group, whereas, C/C (50 %, 56 %) was present in both groups(p = 0.87) (Fig. 3D). Furthermore, among fathers, the T/T genotype was equally present in both groups, but C/C (48.3 %) was seen more in diseased than healthy (p = 0.58) (Fig. 3E). In newborns, C/T (50 %) genotype was mostly present in healthy, whereas, C/C (50 %) and T/T (25 %) were high in the diseased group (p = 0.27) (Fig. 3F).

**Fig. 4:**
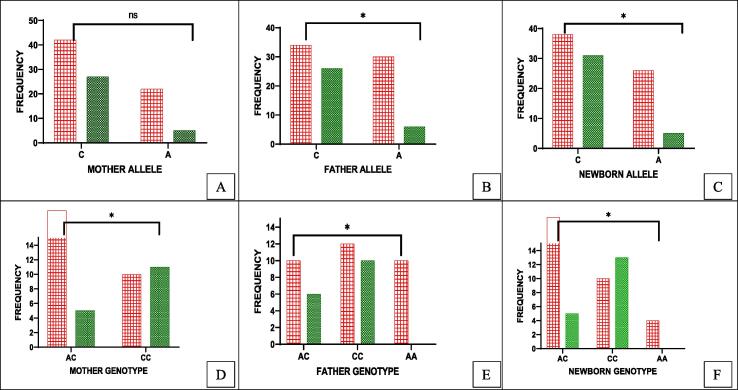
(A-F): Distribution of TERT (rs2736100) allele and genotypes among parents and newborns (triad) in healthy and diseased groups. 4 (A-C): Allele C with high frequency in diseased parents and their newborns. 4D: Diseased mothers with high AC and no AA 4E: CC/AA high in diseased fathers 4F: AC high in newborns to parents with diseases and CC high in the healthy group.

The other TERT gene SNPs (C/A) genotype distribution and polymorphic allele frequency were found statistically significant between all groups: Mother (OR = 2.82, P = 0.08); Father (OR = 3.82, P = 0.01); Newborn (OR = 4.24, P = 0.012) (Table 1). In Fig. 4 (A-C), allele C was more frequent in both diseased and healthy triad samples, whereas, minor allele A was less frequent in the healthy group. However, the A/A genotype was not found in the healthy group of the triad (Fig. 4: D-F). In mothers, C/A (68.8 %) was the most frequent genotype in the diseased group with a significant value (p = 0.03) (Table 2). When the fathers' genotype was assessed again A/A was not found in the healthy group (Fig. 4E) whereas, C/C (65 %) was found with higher frequency, however, in the diseased group A/A (31 %) and C/C (37.5 %) was also present (p = 0.04). Newborns of the diseased group had high C/A (56 %) (p = 0.03). In all groups, HWE was found insignificant (P > 0.5) (Table 2).

Further regression analysis of diseases and newborn TL indicated that hypertension (B = -0.785; CI = -1.554- −0.017; p = 0.045) and diabetes (B = 1.503; CI = -0.130–3.136; p = 0.070) showed significant results than other diseases. (Supplementary Table 1).

**Fig. 5:**
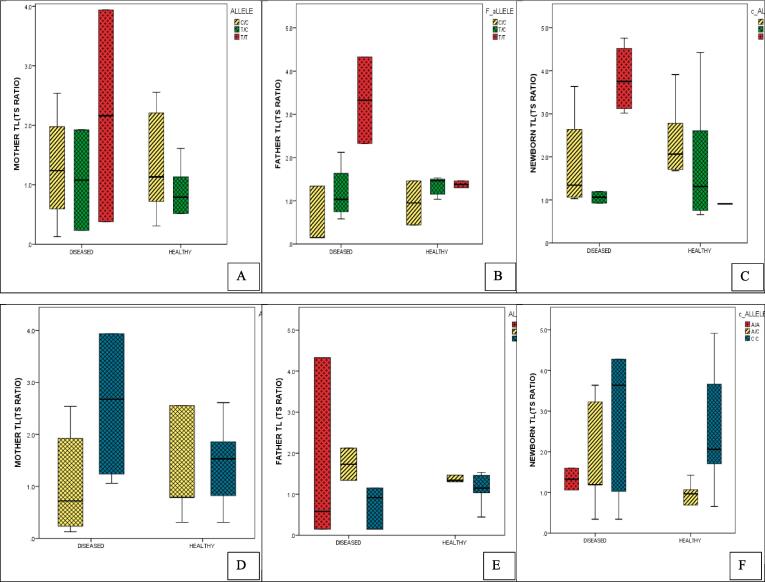
A-F: Comparison of telomere length(TL) (T/S ratio) in TERC and TERT genotypes distribution. 5 (A-C) Longest TL in TERC genotype TT(red bars) among a diseased group of the triad. 5 (D-F) Longest TL in TERT genotypes CC(blue bars) of the diseased group in mother-newborns and AA genotype(red bar) in fathers. (For interpretation of the references to colour in this figure legend, the reader is referred to the web version of this article.)

Comparison of telomere length and TERC genotypes distribution had the longer TL in the healthy group comparing all genotypes. In mothers, the major allele C/C was found with a shorter length (1.28 ± 0.92) (p = 0.32) in the diseased group than in healthy (1.37 ± 0.88) (p = 0.62), whereas T/T was absent in healthy mothers. Analyzing the fathers’ TL and genotypes, C/C and T/C were seen with shorter TL (1.19 ± 0.61, 0.54 ± 0.61) with significant differences (p = 0.032) and longer TL (2.32 ± 1.41, 2.38 ± 0.11) was present in T/T of both diseased and healthy groups. However, in newborns again both T/C and C/C showed shorter TL (1.05 ± 0.18,1.82 ± 0.95) (p = 0.022) in the diseased group, and T/T genotype had longer TL (2.82 ± 2.43) (Fig. 5, Table 3).

**Table 3:** Difference between TERC genotype and Telomere length among triad(n = 48).

**TERC gene**		**Mother**		**Father**		**Newborn**	
Groups	Genotype	TL (T/S ratio) (Mean ± SD)	P value	TL (T/S ratio) (Mean ± SD)	P value	TL (T/S ratio) (Mean ± SD)	P value
Diseased n = 32	**T/C**	1.07 ± 0.97	0.62	1.19 ± 0.61	0.032	1.05 ± 0.18	0.022
	**C/C**	1.28 ± 0.92		0.54 ± 0.61		1.82 ± 0.95	
	**T/T**	2.15 ± 2.05		2.32 ± 1.41		2.82 ± 2.43	
Healthy n = 16	**T/C**	1.89 ± 0.41	0.32	1.34 ± 0.20	0.318	1.80 ± 1.37	0.04
	**C/C**	1.37 ± 0.88		0.95 ± 0.58		2.42 ± 0.94	
	**T/T**	–		2.38 ± 0.11		2.72 ± 0.16	
**TERT gene**							
Diseased n = 32	**A/C**	1.23 ± 0.96	0.03	1.72 ± 0.45	0.11	1.80 ± 1.20	0.00
	**C/C**	1.18 ± 1.56		0.73 ± 0.46		1.71 ± 1.88	
	**A/A**	–		1. 86 ± 2.05		1.32 ± 0.38	
Healthy n = 16	**A/C**	1.39 ± 1.07	0.73	1.36 ± 0.77	0.27	1.96 ± 0.30	0.04
	**C/C**	1.41 ± 0.79		1.12 ± 0.40		2.62 ± 1.54	
	**A/A**	–		–		–	

Krushkal Wallis was applied to study the mean difference among genotypes and telomere length(TL).

For the TERT genotype distribution, the A/A minor allele was not seen in the healthy group in the triad. Similar to the TERC gene, TERT genotypes also had shorter TL in the diseased group compared to the healthy. When the TL and genotypes of mothers and fathers were compared, the C/C genotype was found with shorter TL (1.18 ± 1.56, 0.73 ± 0.46) in the diseased group than the healthy. Whereas, A/A was absent in mothers but in fathers, it had the longer TL (1. 86 ± 2.05) (p = 0.11). Among newborns, C/C had shorter (1.71 ± 1.88) and A/C had longer TL (1.80 ± 1.20) (p = 0.00) (Fig. 5, Table 3).

Regression analysis of parents' genotypes and newborn TL indicated that TERC SNPS contributes insignificantly to the univariate model (P < 0.25). However, for TERT, CC genotype was found significant contributing factor for Newborn TL (B = 0.575; CI = -0.306–1.455; P = 0.196), (B = -0.739; CI = -1.545–0.067; P = 0.071) (P < 0.25) (Supplementary Table 2)*.*

## 4 Discussion

To the best of our knowledge, this study on telomere variation represented for the first time in both parents and their newborns. The polymorphic allele distribution and its association with common diseases highlighted genetic variation. The TERC (rs10936599) SNP (C/T) having minor allele (T/T) with longer telomere length (TL) and major allele (C/C) with shorter TL was seen in the newborns and their parents. Among the diseased group (mother: Gestational Diabetes Mellitus(GDM) and Preeclampsia, fathers: Diabetes and Hypertension) genotype C/C with shorter TL (1.82 ± 0.95)(p = 0.022) was more frequent in newborns. This was in accordance with a study in India which also reported major allele C of the variant, was positively associated with a high risk for type II Diabetes (Odds Ratio = 2.44, *p =* 1.63E-12) (Sethi et al., 2020).

The other targeted gene of the study was TERT, highlighting a minor allele (A/A) present in fathers-newborns of a diseased group with longer TL but the overall C/C major allele in the diseased group was associated with shorter TL also highlighted by other studies (Codd et al., 2013, Snetselaar et al., 2018).

Notably, the TERT gene encodes telomerase enzymes which play a key role in shielding telomere integrity. A *meta*-analysis emphasized positive associates of cancer with the C allele (OR 1.16) and longer telomere length, whereas, a negative association with non-cancerous diseases, (OR 0.81) (Snetselaar et al., 2018). On the other hand, researchers found an A allele associated with shorter TL (AlDehaini et al., 2021) which was not in accordance with this study. Another study (Ye et al., 2017) found the allele “C” as a risk allele in lung cancer susceptibility, this agrees with a longer telomere length vulnerability, therefore, our finding suggests that the TERT locus with a shorter TL in the C allele especially in newborns not prone to cancer development. A study among the Chinese population showed the allele “C” as a minor allele (Codd et al.,2013) in contrast to the current study, results showed the “A” allele as a minor allele among the Pakistani targeted population of this study.

This study explored for the first time parents diseases and their effect on newborn telomere genetics. It was found that diseases in fathers like Diabetes and Hypertension showed an association with newborn TL, which highlighted more influence of fathers' diseases on newborns than mothers. Moreover, telomeres have been extensively studied in association with the risk of different diseases like malignant tumors (Zhu et al., 2014), cardiovascular disease (D'mello et al., 2015), metabolic disease (Révész et al., 2014), upper respiratory track diseases in children (Cohen et al., 2013), and Chronic Pulmonary Obstructive Disease (COPD) (Berndt, et al.,2012).

Data regarding the association between the different common diseases in the triad as a whole with TL and TERC, TERT SNPs is not available. In this study, TL, TERC, and TERT SNPs were found significantly associated with diseases. In the diseased group, the C/C SNP was seen with shorted TL, whereas in the healthy group longer TL was observed. Aldehaini in 2021 observed the shortening of TL in T2DM and found TERT C/A SNP associated with high plasma levels of the telomerase but they did not find any association with telomerase deficiency or TERC and TERT gene polymorphisms (AlDehaini et al., 2021).

However, different studies have correlated TL or telomerase genes (TERC and TERT) polymorphisms with demographics, tumors, chronic cardiovascular problems, and type 2 diabetes (Li et al., 2021, Zhang et al., 2017, Khalangot et al., 2020). Probably these studies were done in chronic development stages, and the results were very significant, showing a correlation between TL, telomerase genes, and the morbidity of different chronic conditions (Zhang et al., 2017).

The diseases discussed in this research and their significant difference with genotypes and TL will add valuable data to the literature. The decrease in TL in the C/T allele of TERC and the A/C allele of TERT in the diseased group compared to the healthy, especially in newborns is supported by a study by Cui et al (Cui et al., 2022). Similarly, a study in China also observed shorter TL with minor allele T in both genes (Weng et al., 2016). It was also found previously that A/C polymorphism was frequently high in the population that has a strong risk of diseases like “idiopathic pulmonary fibrosis (IPF)”, and not for other lung diseases like “interstitial lung diseases”. Therefore, participants of the A/C genotype in this study may be at risk of developing the above diseases. The TERT A/C was seen in hypertension patients having significantly shorter LTL (0.98 ± 0.98 vs 1.76 ± 1.75, P = 0.003), which may result from defective TERT (Cheng et al., 2020).

In the respective study, the regression model confirmed TERT C/C as a major allele and significant contribution factor for newborns. The minor allele “T” of TERC and the “A” of TERT represent the risk allele with longer TL. This was not in accordance with studies highlighting the dominant model of the T/A allele (Codd et al., 2013, Mitchell et al., 2018).

The underlying mechanism of decreased TL is due to the acute stress of reactive oxygen species in different diseases. As the disease progresses, telomere attrition reaches an extremely short length and undergoes apoptosis or senescence and overt to mortality. Another mechanism that leads to cancer induces upregulation of the TERT gene aimed to preserve TL which may cause uncontrolled elongation thus making the cells immortal (Liu et al., 2019).

The TERC C/T allele association with TL showed an increased risk of colorectal cancer (longer TL) (Jones et al., 2011), idiopathic pulmonary fibrosis (shorter TL) (Sousa et al., 2019) and multiple sclerosis (longer TL) (Hecker et al., 2021). Thus it can be inferred from the above data that newborns in this study with longer TL and T/T alleles (2.82 ± 2.43) may be at risk of developing such diseases later in life.

It is well known that TL is a marker of biological aging that maximizes at birth and reduces with advancing age (Martens et al., 2020). In this study newborn, TL was positively correlated with parental TL (r = 0.42,0.39) and had longer TL than parents. However, mothers had more correlation than fathers. The data can be supported by our previous study which also showed a correlation (r = 0.395) between the mother-newborn and longer newborn TL was found than mother TL, thus, highlighting telomere reprogramming (Farrukh et al., 2019, Farrukh et al., 2022). A study elucidated it as a heritability factor and found a similar positive correlation (*β* = 0.14, *P* = 1.99E − 05) (Chen et al., 2022). This emphasizes the hypothesis that during fetal life, TL reprograms in newborns although mothers had a shorter length as highlighted above.

The strength of this study highlighted that maternal, as well as paternal health free of diseases, is a crucial determining factor of TL, which could impact newborn health. Hence, improving the health of parents by altering the targeted modifiable factors (SES, lifestyle), can help in preventing telomere attrition and boost cellular longevity.

The study limitations were the small sample sizes and limited telomerase gene restriction sites explored by Sanger sequencing due to budget constraints. Moreover, there may be a recall bias during the filling of the questionnaire and participants recalling their history.

Therefore, the identification of genes [TERT(C/A), TERC (C/T)] polymorphism and its association with TL emphasized its importance in cellular fate and the role of its length in determining telomere function. Such research can be the first step in interpreting the telomere role and regulatory mechanism in the pathogenesis of diseases.

## 5 Conclusion

There was a positive association observed between newborn Telomere Length (TL) and telomerase genes (TERC, TERT) variants in different diseases of parents. Both genes of telomerase have major allele C, associated with shorter TL in diseased parents of the targeted population, however, the longer TL was seen in minor alleles (T and A). Therefore, telomeres may act as a marker of biological aging and play a significant role in diseases associated with telomere genetics. Future studies including larger sample size and other risk factors must be included to improve the quality of life and aging in parents and their newborns.

## Funding

This research won a grant from the “Higher Education Commission of Pakistan (HEC) National Research Program for Universities- NRPU (Ref No. 20-15896/NRPU/R&D/HEC/2021)”. The Ziauddin University, Karachi Pakistan also funded this research (Ref no. Biochemistry.242.14/5/21).

## Declaration of competing interest

The authors declare that they have no known competing financial interests or personal relationships that could have appeared to influence the work reported in this paper.
