# Supplementary material for: Identification of polymorphic alleles in TERC and TERT gene reprogramming the telomeres of newborn and legacy with parental health
Source: Saudi J Biol Sci. 2023 Dec 12;31(2):103897. doi: 10.1016/j.sjbs.2023.103897 (PMC10772381; doi:10.1016/j.sjbs.2023.103897)
Supplement: Supplementary data 1 [file mmc1.docx]

**Supplementary Data**

Supplementary Table 1: Multivariate Linear Regression Analysis for Newborn TL (T/S Ratio) With diseases

|  | **Univariate Analysis** | | | | **Multivariate Analysis** | | | |
| --- | --- | --- | --- | --- | --- | --- | --- | --- |
| Associated factors | Coefficient  B | 95% Confidence Interval | | P-value | Coefficient  B | 95% Confidence Interval | | P-value |
|  |  | Lower Bound | Upper Bound |  |  | Lower Bound | Upper Bound |  |
| Disease Status | .219 | -.277 | .715 | .386 |  |  |  |  |
| Preeclampsia | -.474 | -1.248 | .300 | .282 | -.232 | -3.361 | 2.897 | .880 |
| Hypertension | -.785 | -1.554 | -.017 | .045* | -1.156 | -3.759 | 1.447 | .371 |
| Gestational Diabetes Mellitus | .219 | -.221 | .658 | .327 |  |  |  |  |
| Diabetes | .561 | -.052 | 1.174 | .073* | 1.503 | -.130 | 3.136 | .070 |
| Significance level: P<0.25(for Univariate linear regression), P<0.05 (for Multivariate linear regression). * significant results | | | | | | | | |

Supplementary Table 2: Multivariate Linear Regression Analysis for Newborn TL (T/S Ratio) With TERC and TERT gene.

|  | **Univariate Analysis** | | | | **Multivariate Analysis** | | | |
| --- | --- | --- | --- | --- | --- | --- | --- | --- |
| Associated factors | Coefficient  B | 95% Confidence Interval | | P-value | Coefficient  B | 95% Confidence Interval | | P-value |
|  |  | Lower Bound | Upper Bound |  |  | Lower Bound | Upper Bound |  |
| TERT gene | | | | | | | | |
| Mother AC | -.110 | -.943 | .723 | .793 |  |  |  |  |
| Mother -CC | .575 | -.306 | 1.455 | **.196*** | .030 | -1.202 | 1.263 | .961 |
| Father- -AA | .436 | -.673 | 1.544 | .434 |  |  |  |  |
| Father -AC | .110 | -.723 | .943 | .793 |  |  |  |  |
| Father -CC | -.739 | -1.545 | .067 | **.071*** | -.719 | -1.865 | .426 | .213 |
| Newborn -AA | .181 | -1.405 | 1.767 | .820 |  |  |  |  |
| Newborn -AC | .038 | -.803 | .878 | .929 |  |  |  |  |
| Newborn -CC | -.086 | -.917 | .744 | .836 |  |  |  |  |
| TERC gene | | | | | | | | |
| Mother-CC | -.018 | -.874 | .837 | .966 |  |  |  |  |
| Mother-TC | -.014 | -.864 | .837 | .974 |  |  |  |  |
| Mother-TT | .061 | -1.116 | 1.238 | .918 |  |  |  |  |
| Father-CC | -.416 | -1.298 | .466 | .348 |  |  |  |  |
| Father-TC | .303 | -.540 | 1.145 | .474 |  |  |  |  |
| Father-TT | .182 | -1.148 | 1.511 | .785 |  |  |  |  |
| Newborn-CC | .120 | -.726 | .966 | .777 |  |  |  |  |
| Newborn-TC | .072 | -.863 | 1.008 | .877 |  |  |  |  |
| Newborn-TT | -.315 | -1.434 | .804 | .574 |  |  |  |  |
| Significance level: P<0.25(for Univariate linear regression),  P<0.05 (for Multivariate linear regression). * significant results | | | | | | | | |
